# Metal-Assisted and Microwave-Accelerated Decrystallization: An Alternative Approach to Potential Treatment of Crystal Deposition Diseases

**DOI:** 10.23937/cabjd-2017/1710002

**Published:** 2017-10-13

**Authors:** Nishone Thompson, Zainab Boone Kukoyi, Carisse Lansiquot, Salih Toker, Bridgit Kioko, Hillary Ajifa, Chinenye Nwawulu, Oluseyi Daodu, Kadir Aslan

**Affiliations:** Department of Civil Engineering, Morgan State University, USA

**Keywords:** Gout, Crystal deposition diseases, Gold nanoparticles, Medical microwaves

## Abstract

Gout is a painful and prevalent crystal deposition disease caused by the overproduction of Uric Acid (UA) in the body and the atypical deposition in human synovial joints as Monosodium Urate Monohydrate (MSUM). Conventional treatments, such as NSAIDs, cyclooxygenase-2 inhibitors, and systemic glucocorticoids often present harmful side-effects and are short-lived. Long-term therapies including xanthine oxidase inhibitors and the use of uricosuric agents have been developed and aim to lower the UA serum levels in the body. As regards to post-crystals deposition, our research laboratory recently proposed and demonstrated the use of the Metal-Assisted and Microwave-Accelerated Decrystallization (MAMAD) technique for the breakdown of organic and biological crystals on planar surfaces. The MAMAD technique is based on the combined use of microwave heating and Au NPs in solution. The interactions of the Au NPs with microwave’s electromagnetic field result in an increase in the kinetic energy of Au NPs, and subsequently, an increase in the collisions with target crystals placed on planar surfaces leading to rapid crystal breakdown. In this regard, our laboratory aims to develop the MAMAD technique as an alternative treatment for crystal deposition diseases, particularly gout, with minimal invasion and side-effects as compared to current treatments. In this review article, we will summarize our previous findings and provide additional data detailing the effectiveness of the MAMAD technique as a rapid and efficient method for the breakdown of gout related crystals and L-alanine crystals (a model crystal).

## Background

### Crystal deposition diseases and gout

The human body hosts complex biological mechanisms in the excretory, salivary, and urinary systems, where activation and inhibition of crystal formation are regulated. Several critical chemical compounds, such as, citrate, pyrophosphate, magnesium, osteopontin and bikunin are employed to prevent crystal nucleation, crystal growth, and/or crystal aggregation in the human body [1]. Malfunctions in these regulatory mechanisms can lead to abnormal fluctuations in the chemical environment, thereby promoting excessive crystallization and atypical deposition in various areas of the body [1,2]. Several human diseases are associated with this aberrant crystal deposition. For example, atherosclerotic plaques and gallstones are formed when cholesterol crystallizes in the arteries and liver, respectively. Kidney stones are the product of uncontrolled growth and nucleation of calcium containing crystals. High concentrations of urate may lead to the formation of needle-like MSUM crystals, which precipitate into the joint cavities producing gouty arthritis [1,2].

Gout is one of the most common form of crystal deposition diseases that is becoming increasingly prevalent worldwide, characterized by the deposition of MSUM in synovial fluid, surrounding tissues and causes excruciating pain [3,4]. Of the adults in developed nations, at least *1%* to 3% of them are afflicted by the gout disease [3,5]. In the United States, 3% to 5% of individuals between the ages of 45 and 65 are affected by gout [3]. Prevalence of gout in industrialized countries is often associated with lifestyle practices, diet and a gradually aging population; though genetics also play a role in its development [4]. A person suffering from gout often complains of intense pain in joints as well as impaired movement during recreational and daily activities [6].

Biochemically, the major cause of gout is an increase in serum level urate, due to overproduction of UA which is stored in the body as urate and often lead to hyperuricemia. UA is the byproduct of the catabolism of purine rich foods, i.e., red meat, beer and seafood [6,7]. Generally, when serum urate levels are > 6 mg/dL, hyperuricemia and acute gout is diagnosed [4]. When excess purines are catabolized into UA in the presence of sodium at ideal pH and temperatures, urate is combined with sodium and transformed into MSUM a more stable form, released into serum by cells that undergo apoptosis. Excess soluble urate in the blood is usually processed by the kidneys [8,9]. However, a disruption in UA metabolism, whether physical or metabolic, impedes or facilitates the processes that lead MSUM crystals deposition in synovial joints and surrounding tissues [10].

### Conventional and non-conventional treatment of gout

Over the last few decades, several conventional and non-conventional remedies have been developed for the treatment of gout. Conventional methods employ nonsteroidal anti-inflammatory drugs, xanthine oxidase inhibitors (allopurinol), cyclooxygenase-2 inhibitors, colchicine, corticotropin, glucocorticoids and corticosteroids. Though these chemical therapies provide some relief for chronic and acute gout, the relief is temporary [11]. In addition, the effectiveness of current chemical therapies is dependent upon dosing regimens, adverse effect profile, therapeutic use, and toxicity [12]. Studies have shown that adverse side-effects generally develop with the use of the above-mentioned drugs. For example, COX-2 inhibitors were shown to correlate to an increased risk of cardiovascular disease development [12,13]. Nonsteroidal anti-inflammatory drugs have been linked to gastrointestinal toxicity, renal toxicity or gastrointestinal bleeding [14]. Colchicine has been considered a major toxin and may lead to multiple organ failures, convulsions, coma, and death [15]. Glucocorticoid and corticosteroid treatments are affiliated with decreased immune function, osteoporosis, myopathy, avascular necrosis and cardiovascular diseases [16]. Non-drug based therapeutics that have been used as long-term preventative measure involves change in dietary and lifestyle habits [17].

Current non-conventional methods for treatment of gout include plant and herb based home remedies like terpenes, *Angelica dahurlca* (Angelica root) and *Artemisia anomala* (sweet gum fruit) due to their anti-inflammatory properties, lower incidence of allergen city and their active ingredients acts as phytomedicine, a potential replacement for xanthine oxidase inhibitors [18–23]. Xanthine oxidase is a hormone needed for the production of UA from purine nucleotides [24]. The major flaw in the current non-conventional approach is the lack of extensive research in this area and, subsequently, there are no specific dosing for the herbs [25–27]. Due to the limitations of current conventional and non-conventional therapies, an invasive surgery may be required once the disease progresses to chronic stages causing the formation of tophi. Therefore, our research laboratory sought to find an alternate and less invasive method for treatment of tophaceous gout using microwaves, i.e., the MAMAD technique.

### Medical microwave and uses

Microwaves are used in medicine for the diagnosis and treatment of various types of cancer, tissue ablation, the reduction of inflammation and hyperthermia therapies [28–30]. Microwaves applied in non-lethal doses cause the agitation of cells or tissue thereby stimulating a desired and reversible transformation [30]. Microwaves may also cause irreversible transformations when the electromagnetic radiation acts as a stressor with common endpoints in the cell, such as, cellular apoptosis or necrosis [30]. It is also important to note that the frequency of the microwaves and their various penetration depths dictate their efficiency and the type of treatment. For example, electromagnetic radiation of lower microwave frequencies penetrates deeper into biological materials. In this regard, microwaves with lower operating frequencies of 915 MHz and 2.45 GHz are applicable for use in large volume ablation. The difference in the dielectric properties of water and the microwaves make this method suitable for tumor ablation. When exposed to microwave heating, water molecules rotate and produce heat, which induces necrosis in tumor cells [28]. Microwaves at higher frequencies ranging from 5.8-10 GHz produce shallow penetrations and are thereby suitable for near surface based treatments [31]. To avoid damage to biological materials, the frequency of microwaves power the location of electromagnetic radiation and the dielectric properties of the penetrated biological material should be regulated [30].

### The Metal-Assisted and Microwave-Accelerated Decrystallization (MAMAD) technique

#### Proof-of-principle demonstration of the MAMAD technique:

Due to side-effects that arise as a consequence of current therapies and the invasive nature of surgery, finding alternative remedies for gout is an important goal for researchers worldwide. In response to the dire need for new treatments, our research laboratory introduced and developed the MAMAD technique as a potential treatment method for the deposition of tophaceous of UA crystals in joints. Au NPs used as the therapeutic aid in the MAMAD technique is maintained in aqueous solution due to their ability to maintain a surface charge and electrostatic repulsion interactions, which stabilizes Au NPs in the solution against aggregation [32]. The microwaves applied as a therapeutic aid in the MAMAD technique help keep the particles in suspension. The Kinetic energy increase experienced by the particles shows an increased in the collision force induced by the heat generated by the application of microwaves [33]. This article will summarize the results of the MAMAD technique since our initial paper in 2014. We will also include new observations to demonstrate the current advances in the MAMAD technique.

**Figure 1:**
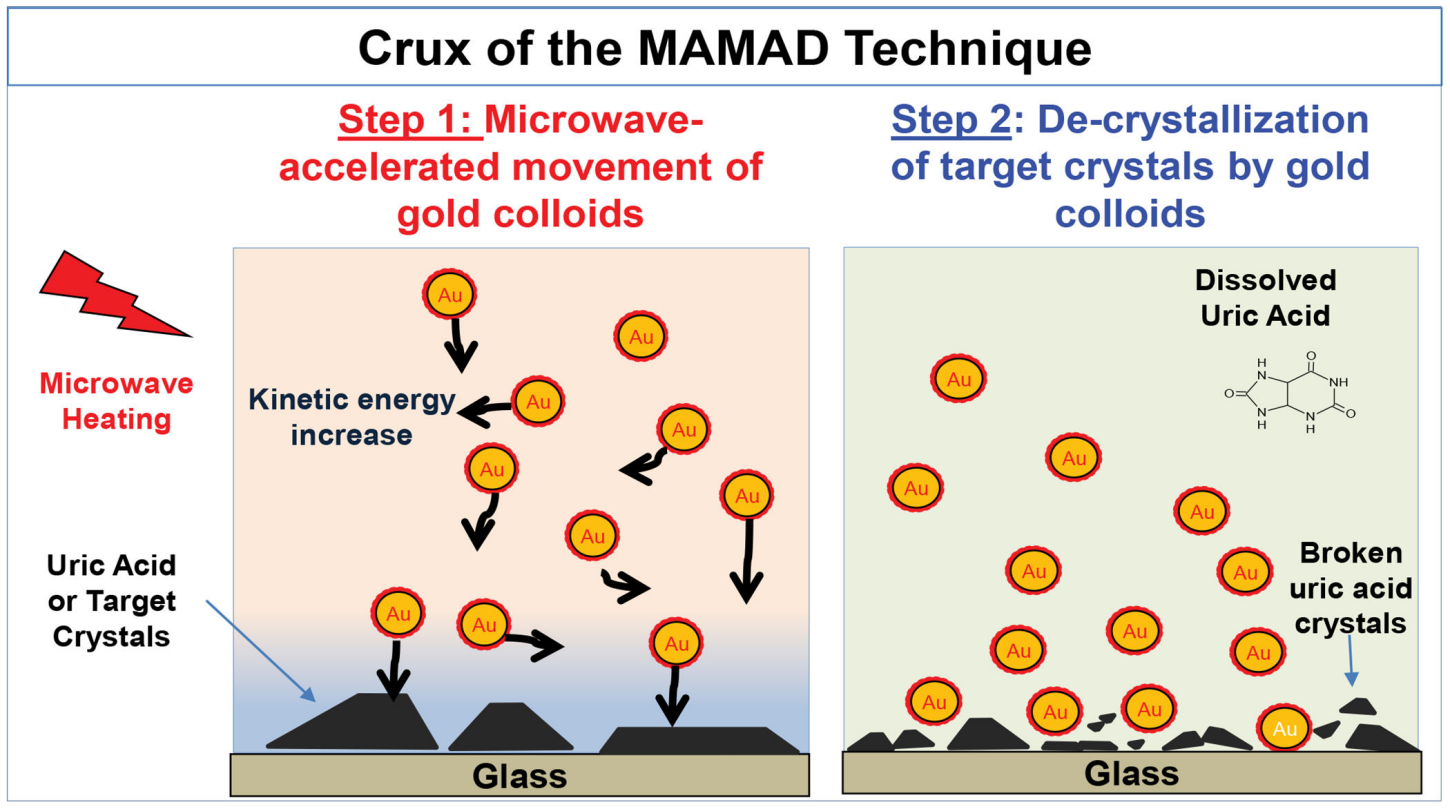
Image depicting the mechanism for the Metal-Assisted and Microwave-Accelerated Decrystallization (MAMAD) technique. Target crystals (i.e., uric acid crystals) are up to 100~ mm and gold colloids are 20 to 200 nm in size.

The MAMAD technique is based on the combined use of Gold Nanoparticles (Au NPs) in solution and the application of low powered direct microwave heating (e.g., 2.45-8 GHz) used for the rapid crystallization and decrystallization of various organic molecules [33,34]. Figure 1 depicts the crux of the MAMAD technique, where: 1) Upon irradiation with microwave energy, the differences in thermal conductivity of the Au NPs, the target crystals and water molecules produces a thermal gradient, 2) Kinetic energy of the Au NPs increases thereby affording the displacement of the Au NPs at high velocities, 3) High speed nanoparticles act as ‘nano bullets’ when they collide with the surface of crystals attached to a solid surface and result in a physical break down of the target crystals [33,35].

In theory, if the MAMAD technique were to be executed *in vivo*, we postulate that the crystal fragments will dissolve into solution, reabsorbed by the body and removed from the body by excretion of excess UA in urine [3]. In terms of the practical application of the MAMAD technique, Au NPs can be injected directly into the affected joints and microwave heating can be locally applied to the area affected by gout. Au NPs are inert and is the only nanomaterial approved by the Federal Drug Administration (FDA) for use as a therapeutic and/or diagnostic agent, and the smaller particles (1-100) are easily eliminate by the liver and kidneys [36,37].

Our research group initially demonstrated the MAMAD technique *in vitro* on a planar glass surface and with Au NPs. The MAMAD technique was used for the rapid decrystallization of UA crystals in a conventional microwave. As the MAMAD technique was advanced, our laboratory demonstrated its effect on the organic and other biological crystals (L-alanine) and MSUM. Furthermore, a new 8 GHz solid-state, monomode medical microwave was implemented in the MAMAD technique as a source of microwave energy and used on artificial joint and skin models. These results are provided in the following sections.

### Decrystallization of uric acid

The proof-of-principle demonstration of the MAMAD technique was carried out for the decrystallization of UA crystals on a planar glass surface [38]. UA is the precursor molecule related to the formation of gout related crystals (i.e. Urate crystals, MSUM crystals), and therefore, is a suitable model molecule for our studies. In a typical MAMAD experiment, decrystallization of UA crystals are carried out on glass slides or on poly(methyl) methacrylate platforms surface which was modified with silane or collagen and silicone isolator wells. Set concentrations and volumes of UA and Au NPs were then placed into wells and covered with a glass micro slide. Microwave energy was applied by placing samples into a conventional microwave oven or with the application of a pen tip solid state monomode microwave. Some of the results in this review article are expressed as retention or normalized values. The retention rate calculated by dividing the value obtained at any given time by the value at time = 0.

**Figure 2:**
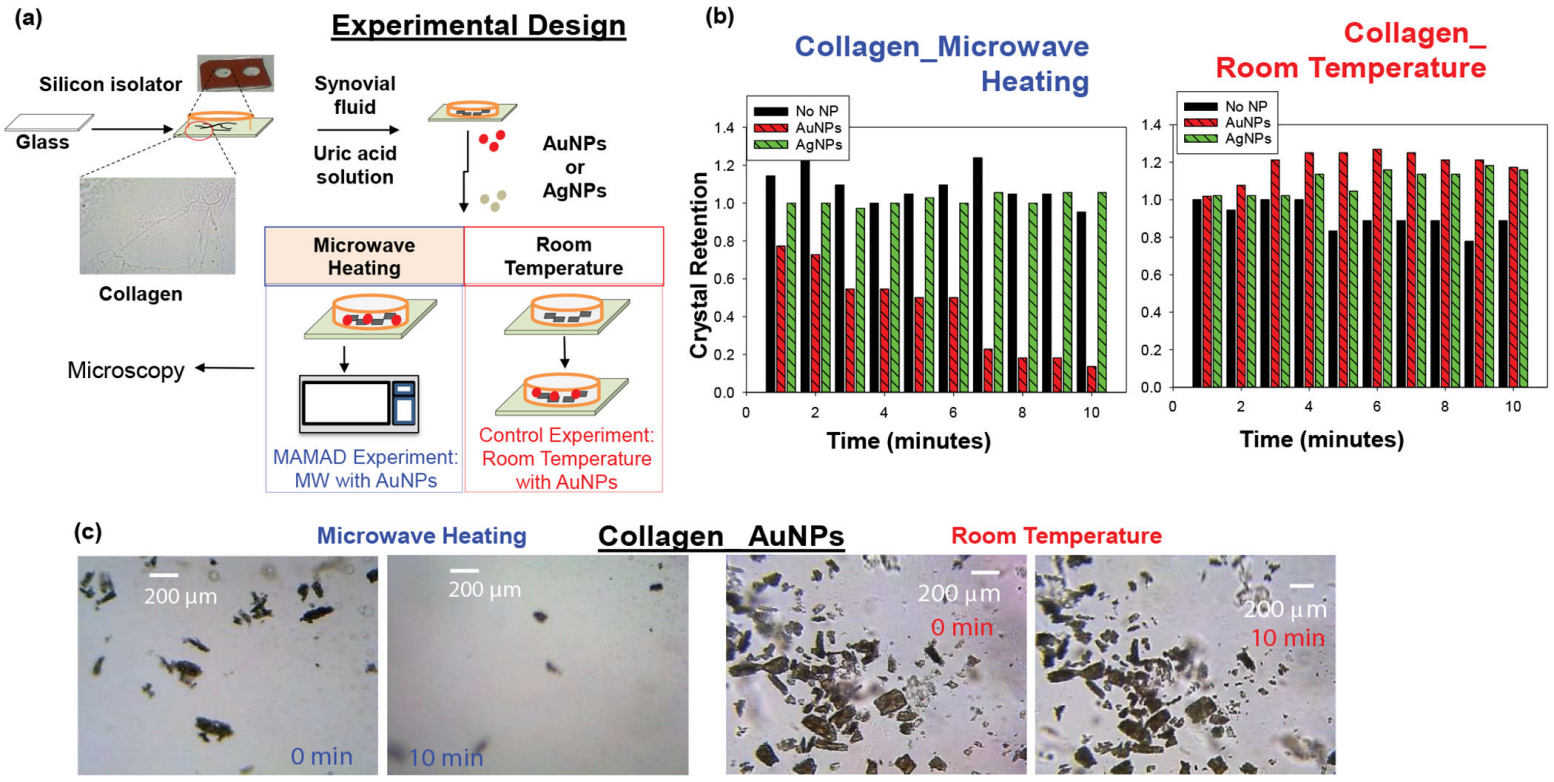
(a) Experimental design for MAMAD technique applied to uric acid on collagen coated glass; (b) Normalized retention of uric acid crystals at room temperature and at microwave temperature on collagen coated glass; (c) Optical images of uric acid crystals at room temperature and microwave temperature before and after 10 minutes.

Details on the specific experimental design for the decrystallization of UA on collagen coated glass and the application of biological fluids that are found in bone joints are illustrated in Figure 2A [34]. In this procedure, UA crystals and synovial fluid were added to a collagen coated glass slide on top of which a silicon isolator and glass cover slip were placed. Note that collagen and synovial fluid were added to the solution to better represent the manifestation of gout in human joints. Finally, Au NPs or Silver Nanoparticles (Ag NPs) were added into the solution and the mixture was microwave heated intermittently in a conventional microwave for 10 minutes or left at room temperature (i.e., a control experiment). Optical images were taken using microscope software and analyzed using ImageJ software for the quantification of the count and size of UA crystals. The effect of using different metal nanoparticles (i.e. Ag NPs and Au NPs) on the UA crystals is explained in the section below [33].

Au NPs and Ag NPs were tested to investigate the most suitable metal nanoparticles for UA breakdown using the MAMAD technique. Analysis of the UA crystals revealed a significant reduction in the number of crystals with the addition of unmodified Au NPs compared to unmodified Ag NPs after exposure to microwave heating. Figure 2B [34] Shows that the number of crystals was reduced by approximately 80% when Au NPs and microwave energy were employed, whereas the use of Ag NPs appeared resulted in no significant changes in the crystal count. This effect can also be visualized in Figure 2C [34], where optical images reveal a dramatic decrease in the crystal number and size after 10 minutes of microwave heating with Au NPs.

**Figure 3:**
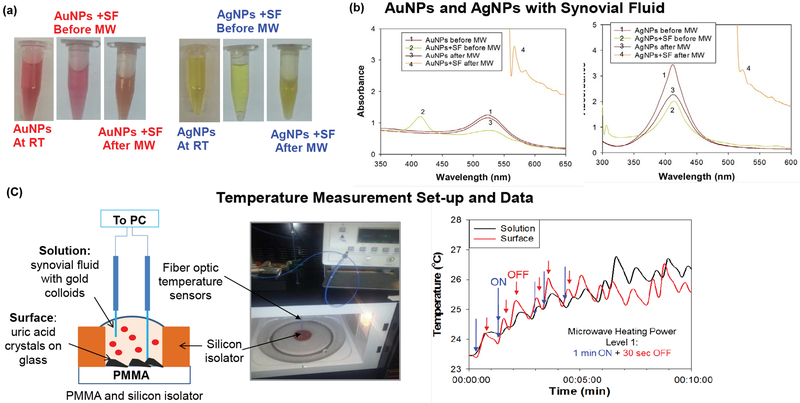
(a) Optical images of silver and gold nanoparticles before and after microwave heating; (b) Real-time temperature of the solution and the surface of the glass slides in a well measured inside a 900 W microwave cavity using fiber optic temperature sensors; (c) Schematic depiction and real-color picture of the experimental setup for temperature measurements inside the microwave cavity.

To investigate the role of metal nanoparticles in the MAMAD technique, real-color pictures and optical absorbance spectra of Au NPs and Ag NPs in the presence of synovial fluid before and after microwave heating were measured and shown in Figure 3A and Figure 3B, respectively. These results showed that Au NPs and Ag NPs remain stable in solution as explained in the following: Optical absorption spectra for Au NPs only and for the Au NPs in synovial fluid before microwave heating showed peaks at similar wavelengths of 520 nm and 532 nm, respectively. Microwave heating of Au NPs alone resulted in no significant change in the absorption spectra of the Au NPs, which implied Au NPs do not aggregate during microwave heating. Microwave heating of Au NPs in synovial fluid resulted in a change in the color of the mixture (to cloudy white and orange), as evidenced by absorption spectra (Figure 3B), which can be attributed to the denaturation of proteins in the bovine synovial fluid. Ag NPs in solution appear to be less stable when microwave heated, where a decrease and broadening of absorption spectrum at 420 nm are observed. A further broadening in the absorption spectrum around 420 nm is also observed when Ag NPs are mixed with a synovial fluid solution. Microwave heating of Silver nanoparticles and synovial fluid solution resulted in a change in color of the mixture (to cloudy white and yellow), which can be attributed to denaturation of proteins in the bovine synovial fluid. In this regard, denaturation of proteins can cause aggregation of Au NPs and Ag NPs. However, the aggregation is more extensive for Ag NPs. Aggregation of metal nanoparticles is unfavorable for the MAMAD technique since the aggregates of metal nanoparticles act as a nucleation site for UA rather than acting as ‘nano bullets’ in motion in the biological media.

Since the MAMAD technique is based on microwave heating of biological samples, it is important to monitor the extent of temperature changes during microwave heating process to determine whether temperature of the media can be maintained near physiological temperature and increase in temperature can be controlled. Figure 3C [34] depicts the experimental set-up used to record temperature measurements throughout the 10 min microwave heating in a conventional microwave oven. As shown in Figure 3C [34], the temperature of the platform (where UA crystals are located and Au NPs solution) during microwave heating increased by ~2.5 and ~3 °C, respectively. Based on these observations, Au NPs were deemed to better act as ‘nano bullets’ for the MAMAD technique. Subsequently, we continue to use Au NPs with the MAMAD technique in our current decrystallization studies.

**Figure 4:**
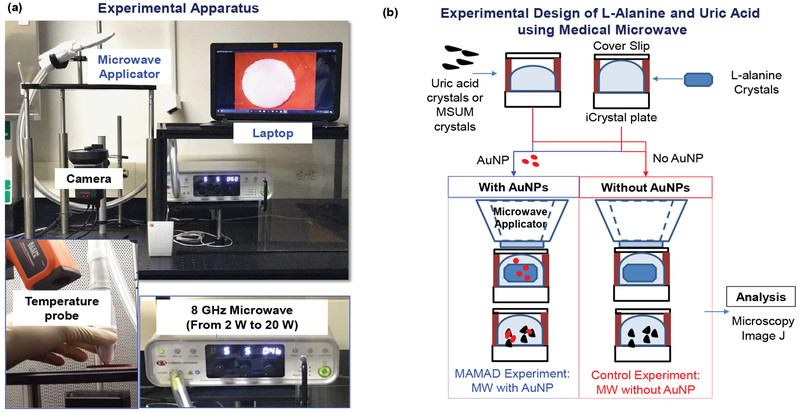
(a) Real color picture of experimental set-up; (b) Experimental design for the decrystallization of L-alanine, MSUM and uric acid crystals using the medical microwave.

Since the use of a conventional microwave oven is not feasible with the MAMAD technique in future applications in humans, a medically approved solid-state microwave source was used to implement the MAMAD technique for the decrystallization UA crystals. As will be demonstrated further along in the text, the medical microwave increases the efficiency of the MAMAD technique by reducing application time significantly. Figure 4A [38] shows the experimental apparatus used throughout the UA and (MSUM) decrystallization experiments. As shown in Figure 4B [38], the MAMAD technique was carried out in a similar fashion as previously described with UA on collagen coated glass.

**Figure 5:**
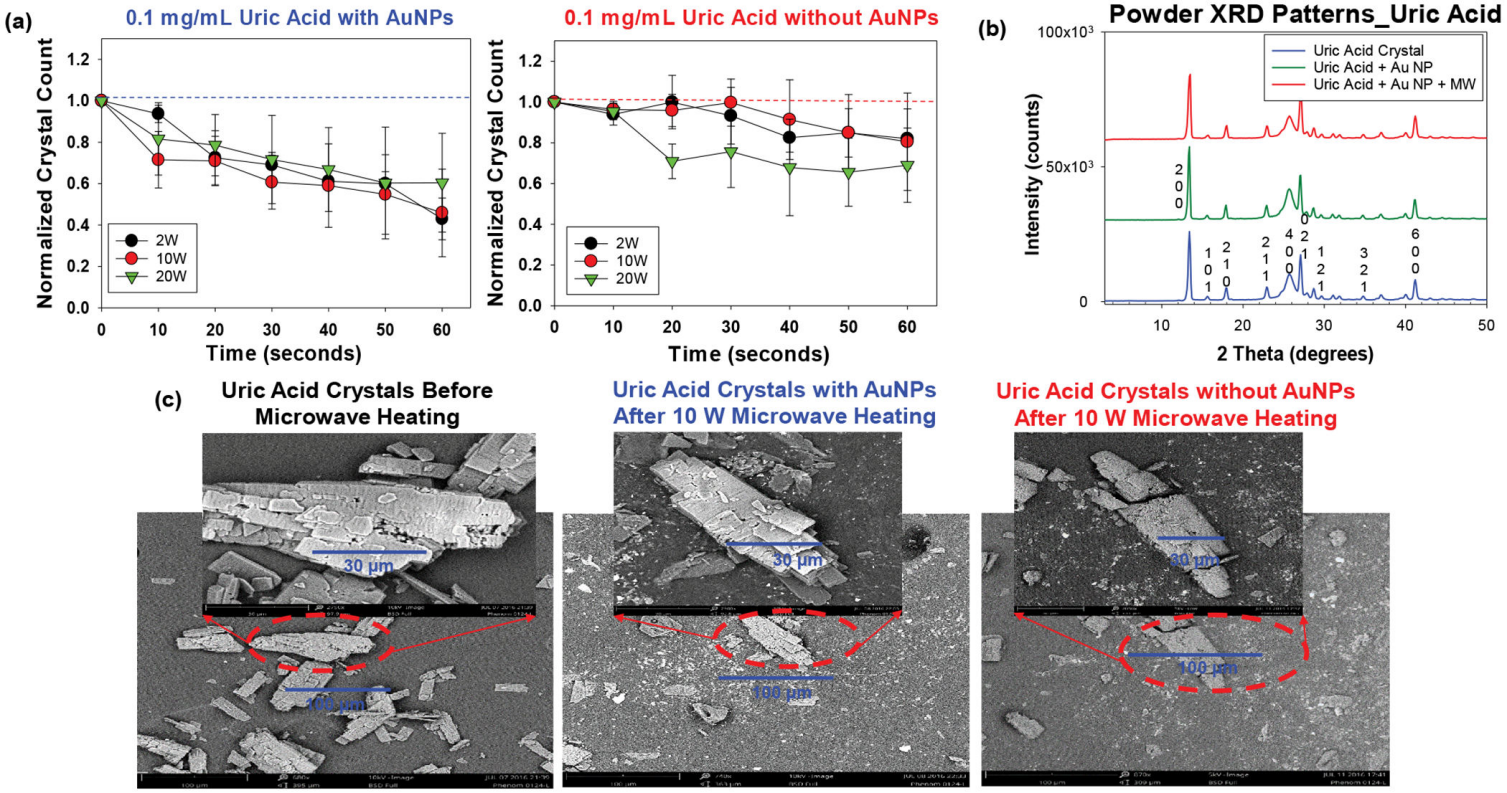
(a) Normalized crystal count of uric acid with and without AuNPs at 2 W, 10 W and 20 W; (b) Powder XRD patterns of uric acid crystals only, without Au NPs and after the application of the MAMAD technique; (c) SEM images of uric acid crystals before and after microwave heating in the presence and absence of gold nanoparticles Au NPs at 10 W.

We sought to determine the best microwave power level for UA decrystallization with the medical microwave, where we observed a decrease in the size and number of UA crystals (Figure 5). Microwave heating at 2 W to 20 W in the presence of Au NPs were used for the decrystallization of UA crystals at 0.1 mg/mL. Figure 5A [38] shows that the 2 W and 10 W power levels decrease crystal count by approximately 57% and 54%, respectively. However, in the control experiments (UA without Au NPs), the extent of decrease for the 2 W and 10 W power level was only 18% and 19% respectively. The reduction in crystal size followed a similar trend with the 2 W and 10 W microwave power levels. A greater extent of solvent evaporation was noted with the 20 W power levels. Based on these observations, 2 W and 10 W power levels were deemed to be most efficient in the reduction of UA crystal size and number at the 0.1 mg/mL concentration.

To investigate the effect of microwave heating on the crystallinity of UA crystals, X-ray crystallography was carried out on UA crystals only, UA crystals with Au NPs only, and UA crystals with Au NPs and microwave heating. Similar X-ray diffraction patterns obtained (no widened peaks or appearance/disappearance of additional peaks) in Figure 5B [38] shows that the crystallinity of the UA remained unchanged in the presence of Au NPs at both room temperature and after exposure to microwave heating. Scanning Electron Microscopy (SEM) images in Figure 5C [38] further support these findings by highlighting that the decrease in sizes obtained was as a result of a physical breakdown and not a change in chemical composition of the crystals. Comparison of the SEM images in Figure 5C proves that the MAMAD technique is efficient in the breakdown of UA crystals as compared to the controls (UA without Au NPs and microwaves and UA crystals at room temperature). This conclusion was reached by the visual confirmation that more fragments of crystals are present in the SEM image of UA using the MAMAD technique. Also, UA crystals are smaller in size and have less defined edges after microwave application as compared to the UA crystals before microwave heating. Once UA crystals are broken in to smaller fragments they are easily dissolved in organic solutions, which will enable excretion in an *In vivo* environment [39]. Increased crystal fragments in tissues usually causes flare-ups in gout cases with increased inflammation, which subsides once smaller crystals are dissolved and reabsorbed [40].

**Figure 6:**
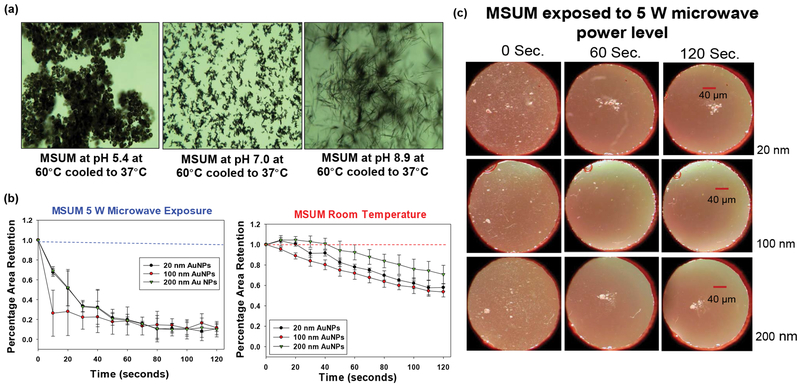
(a) Optical images of different types of Monosodium Urate Monohydrate (MSUM) crystals synthesized experimentally; (b) Normalized retention of MSUM at room temperature and at 5 W power level with gold nanoparticles of sizes 20 nm, 100 nm and 200 nm; (c) Optical images of MSUM crystals before and after 5 W microwave heating and with Au NPs of sizes 20 nm, 100 nm and 200 nm.

Our research laboratory also aimed to demonstrate the use of the MAMAD technique for the decrystallization of other crystals that resemble the actual biological crystals found in gout patients. Consequently, we produced MSUM crystals *In vitro* under controlled temperatures and pH conditions. Figure 6A shows optical images of the various types of MSUM crystals that were produced in our laboratory. As the pH increased, the morphology of the MSUM crystals also changed. MSUM crystals grown at a pH of 8.9 were selected due to their shape, which matched the crystals formed in the synovial joints of the body [41]. In addition, the effect of the size of Au NPs on the MAMAD technique was also investigated. Subsequently, different sizes of Au NPs (20 nm, 100 nm and 200 nm) were used in the decrystallization process using microwave heating at 5 W and at room temperature (control) as seen in Figure 6B. In both scenarios, the decrease in the size of MSUM crystals was measured by using ImageJ software. There was no significant difference between the reductions in the surface area occupied by the crystals when using 20 nm, 100 nm or 200 nm Au NPs. The use of all three Au NPs sizes resulted in a decrease in the surface area occupied by the MSUM crystals by ~90% within 120 seconds of microwave heating. Figure 6B also shows that 20-40% of the MSUM crystals were decrystallized at room temperature, which was considerably less as compared to the values observed using the MAMAD technique. Based on these results, 20 nm Au NPs were selected for further use due to their smaller size and physical stability in biological solutions as compared to the other Au NPs sizes. Figure 6C shows images of the MSUM crystals before, during and after microwave heating obtained using an optical microscope: An initial picture was taken at 0 seconds, one at 60 seconds and a final picture at 120 seconds. These optical images also corroborate that the extent of decrystallization is similar for all sizes of Au NPs.

### Decrystallization of L-alanine crystals (a model tophi)

**Figure 7:**
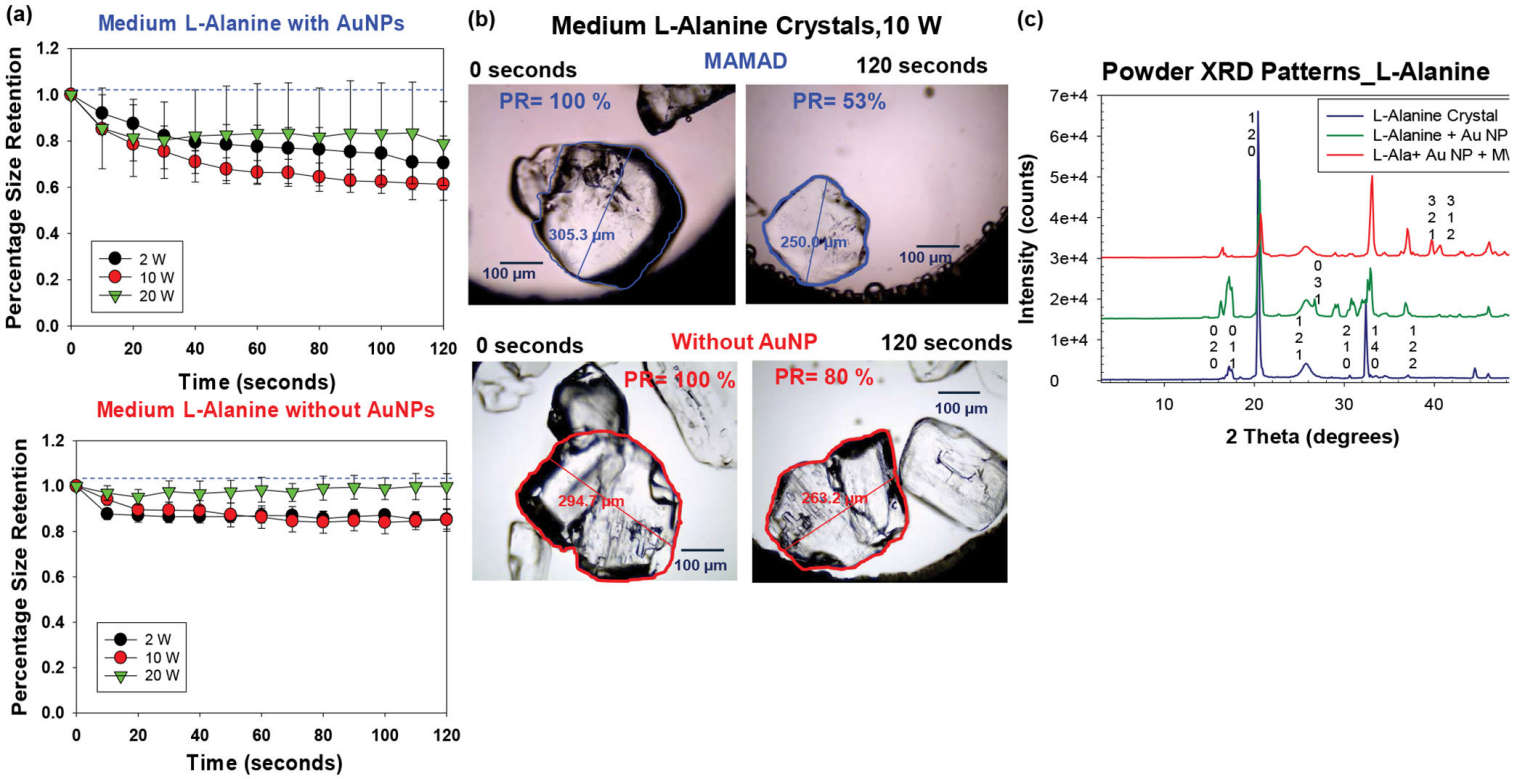
(a) Normalized size retention of L-alanine crystals with and without Au NPs at 2 W, 10 W and 20 W; (b) Optical images of L-alanine crystals after microwave heating at 10 W for 120 seconds in the presence and absence of gold nanoparticles Au NPs; (c) Powder XRD patterns of L-alanine crystals only, without Au NPs and after the application of the MAMAD technique.

Although the MAMAD technique was demonstrated to break down the UA and MSUM crystals, the size of these crystals (< 100 μm) are significantly smaller than tophaceous crystals found in gout patients (in millimeter scale). In this regard, L-alanine crystals, which are much larger (up to 4.5 mm), were selected as a more ideal model for tophaceous gout. The experimental design for the decrystallization of L-alanine crystals using the MAMAD technique is shown in Figure 4B [38]. In a control experiment, L-alanine crystals without Au NPs are exposed to microwave heating. The purpose of these experiments was to compare the effect of microwave power level on the decrystallization of large crystals with the small crystals using the MAMAD technique. Figure 7A [38] shows that the use of 10 W power resulted in ~40% decrease in the size of L-alanine crystals, thus proving to be the most effective microwave power level for L-alanine decrystallization as compared to a control experiment and the other power levels. The use of 2 W and 20 W of microwave power only provided a decrease of ~30% and 20%, respectively. However, the use of all microwave power levels resulted in decrystallization of L-alanine crystals as compared to the control experiments, which provides direct evidence for the effectiveness of the MAMAD technique for the breakdown of large molecules in the millimeter-scale. Figure 7B [38] provides a visual evidence for the decrystallization of the L-alanine crystals during microwave heating for 120 seconds and in the presence and absence of Au NPs. Using the MAMAD technique, there is a decrease of ~47% in L-alanine crystal size, which is significantly higher than that observed in the control experiment (~20%). Evaporation of solvent is more pronounced when microwave heating at 20 W is employed, which is thought to reduce the efficiency of decrystallization using the MAMAD technique. However, a tradeoff between microwave power and decrystallization efficacy is made when lower microwave power is chosen: Although decrystallization of larger L-alanine crystal requires higher microwave power level, the use of lower microwave power level is expected to result in less damage in biological samples.

Figure 7C [38] shows the X-ray crystallography patterns of L-alanine crystal only, L-alanine and Au NPs and L-alanine with Au NPs and microwave heating, which reveal that the crystal structure of L-alanine is not altered, as demonstrated by the identical the XRD patterns, proving that the crystallinity of L-alanine is unchanged. When the MAMAD technique is used, additional faces at (321) and (312) for L-alanine appear as pieces of the crystal are broken away by Au NPs due to microwave heating. Similar observations were made with the X-ray diffraction patterns of UA, thereby proving the effectiveness of the MAMAD technique in the decrystallization of both small and large crystals. It is important to note that the MAMAD technique does not affect the polymorphism of the crystal structures, but instead breaks them down into smaller pieces.

### MAMAD technique and decrystallization of crystals using a pouch model and synthetic skin

**Figure 8:**
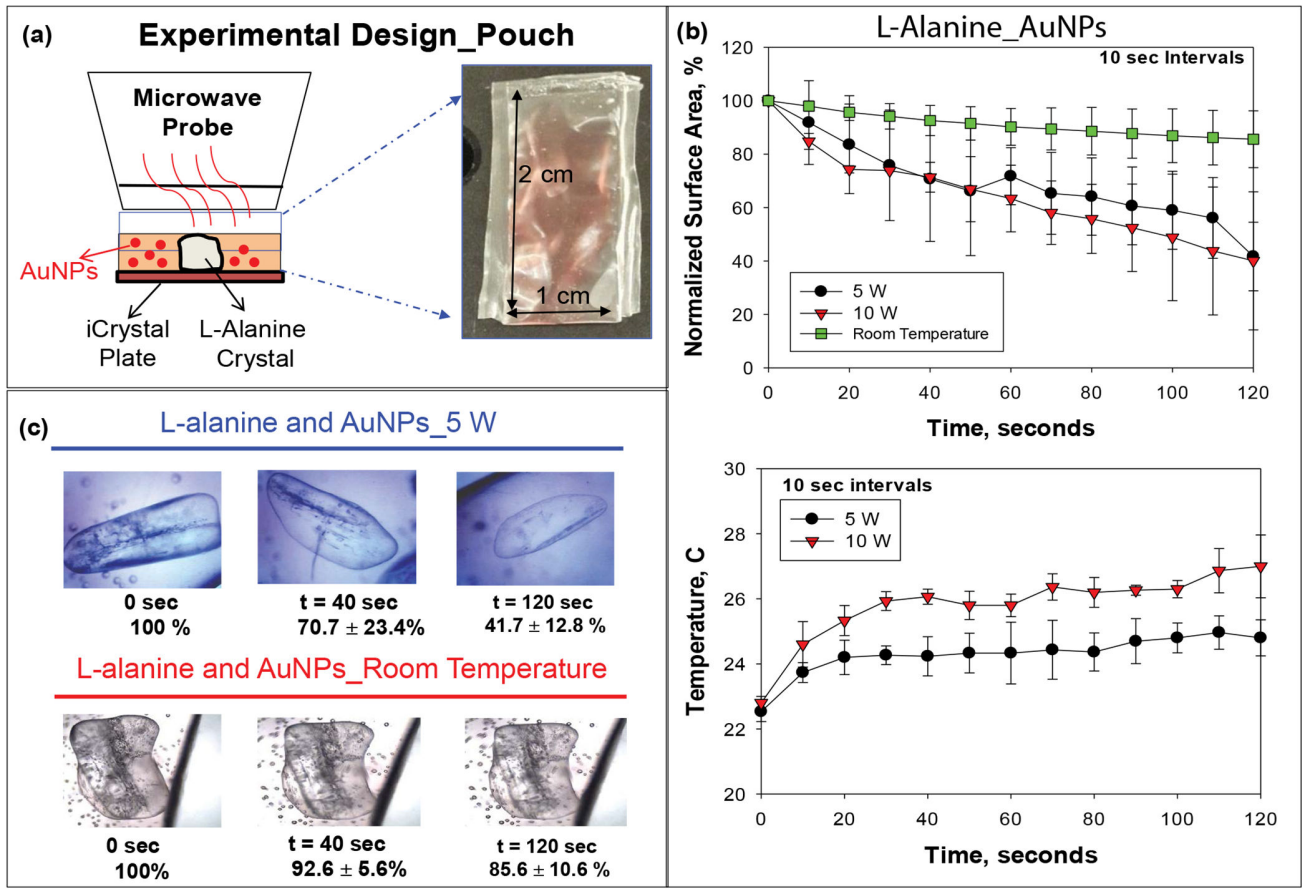
(a) Experimental design of experiment with L-alanine in pouch; (b) Normalized surface area and temperature changes of L-alanine crystals in pouch at room temperature or heated at 5 W and 10 W; (c) Optical images of L-alanine crystals in pouch heated at 5 W and at room temperature.

To get a broader perspective on the potential applicability of the MAMAD technique in mammals, we created a more sophisticated tophaceous gout model that utilizes a synthetic pouch (i.e., plastic). The synthetic model mimics the accumulation of MSUM in the synovial fluid which occurs prior to the formation of tophaceous mass formation, which is presented in the advance stages of gout [42]. The plastic pouch used is representative of the synovial membrane that encases the joint cavity in which UA crystals deposit in humans. Both the synovial membrane and the synthetic pouch represent a physical barrier and hypothetically present similar outcomes when the MAMAD technique is applied to crystals enclosed within. Figure 8A depicts a visual representation of the pouch model design and the dimensions of the pouch (2 cm × 1 cm). The microwave power level was set to 5 W and applied in increments of 10 seconds for a total of 120 seconds. The surface area of the L-alanine crystals before, during and after microwave heating was obtained by outlining the surface of each crystal using ImageJ software. Figure 8B shows that the use of the MAMAD technique at 5 and 10 W results in up to 60% reduction in the average surface area of L-alanine crystals. In the control experiments (L-alanine in pouch with Au NPs at room temperature i.e., no microwave heating), the average surface area of L-alanine decreased by ~14%. Temperature of the solution increased ~2 °C and 4 °C for the 5 W and 10 W powers respectively. Optical images shown in Figure 8C provide a visual evidence for the reduction of size of the L-alanine crystals using the MAMAD technique. It is also important to monitor the temperature of the solution where Au NPs and L-alanine crystals are present during microwave heating. These results imply that the use of microwave heating 5 W and 10 W will cause insignificant damage to the synthetic membrane while the crystals can be decrystallized in a rapid manner.

**Figure 9:**
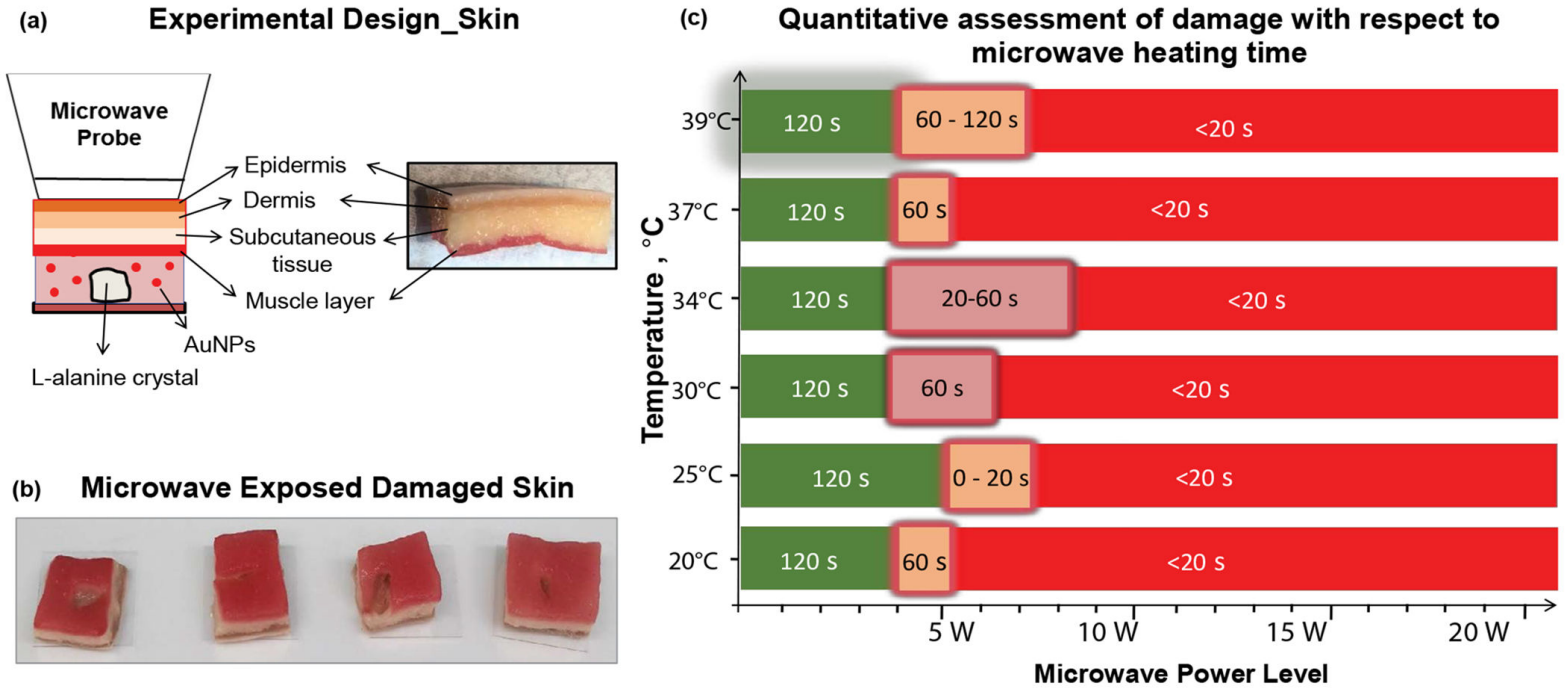
(a) Experimental design of L-alanine under skin; (b) Images of damaged skin when exposed to microwave energy; (c) Quantitative assessment of skin damage with respect to microwave heating time. Green scale denotes the time and microwave power where no tissue damage was observed in the synthetic skin (note that the initial temperature of the synthetic skin was adjusted to the values given in y-axis). Glowing orange scale is “transition zone” where minor tissue damage was observed. Red scale denotes significant tissue damage. Time values indicate the duration of microwave heating where the synthetic skin remains undamaged.

Although the MAMAD technique was demonstrated to work efficiently with a synthetic membrane, certain biological factors were still missing from our experimental setup. For example, *In vivo*, the joint consists of skin in addition to a cavity and membrane. Subsequently, we investigated a skin model (synthetic skin) to ascertain potential quantitative and qualitative damage and temperature changes during microwave heating of synthetic skin. Figure 9A [31] depicts the experimental set-up, where a single large L-alanine crystal and Au NPs solution is placed inside a silicon isolator well and is covered by a synthetic skin sample consisting of several layers. Synthetic skin samples are comprised of epidermis, dermis, subcutaneous tissue and muscle layer [31]. Microwave heating of the synthetic skin samples was carried out intermittently for 120 seconds with 20-second intervals, and real-color images were taken for qualitative assessment. It is important to note that the synthetic skin is of similar texture and consistency to the average human skin tissue and its puncture pressure of 2.0 N/mm^2^ is like the puncture pressure of actual human skin (2.5 ± 0.3 N/mm^2^).

To further investigate the effect of initial temperature of the synthetic skin samples, experiments carried out with the synthetic skin samples were kept at various initial temperatures (range was from 20 °C to 39 °C). Initial temperatures below physiological temperature of 37 °C are used to simulate conditions in which the gout patient is placing ice on skin for comfort. Temperatures at near about physiological temperature are used to simulate conditions of inflammation where the temperature of the skin rises. Potential damage to both the upper and lower portions of the synthetic skin samples was evaluated. Visual evaluations of the synthetic skin samples before and after microwave heating (not shown in this article) demonstrate that microwave power ranges of less than 4 W negligibly affect the top and bottom surface area of the synthetic skin samples, regardless of the initial temperature. However, above the 5 W power level, qualitative analysis show that significant damage, such as, tears or burning of the lower surface of the synthetic skin occurs. The extent of the damage after microwave exposure is highlighted in Figure 9B [31]. Quantitative calculations of the damage to the synthetic skin samples were also done by measuring the surface areas of the bottom and top of the sample before and after 20 seconds of microwave exposure using ImageJ software. Figure 9C [31] corroborates the qualitative data obtained from the real color images with a summary of the quantitative findings. According to Figure 9C [31], synthetic skin samples can safely be exposed to 4 W for up to 120 s without significant structural damage to the synthetic skin, regardless of the initial temperatures (green zone). For an additional 20-120 seconds, a maximum of 7 W can be tolerated by the synthetic skin samples, though some damage is experienced (orange zone). Above 7 W microwave exposure however, the synthetic skin samples are significantly less stable (red zone) after exposures lasting 120 s (50% decline in stability is predicted).

**Figure 10:**
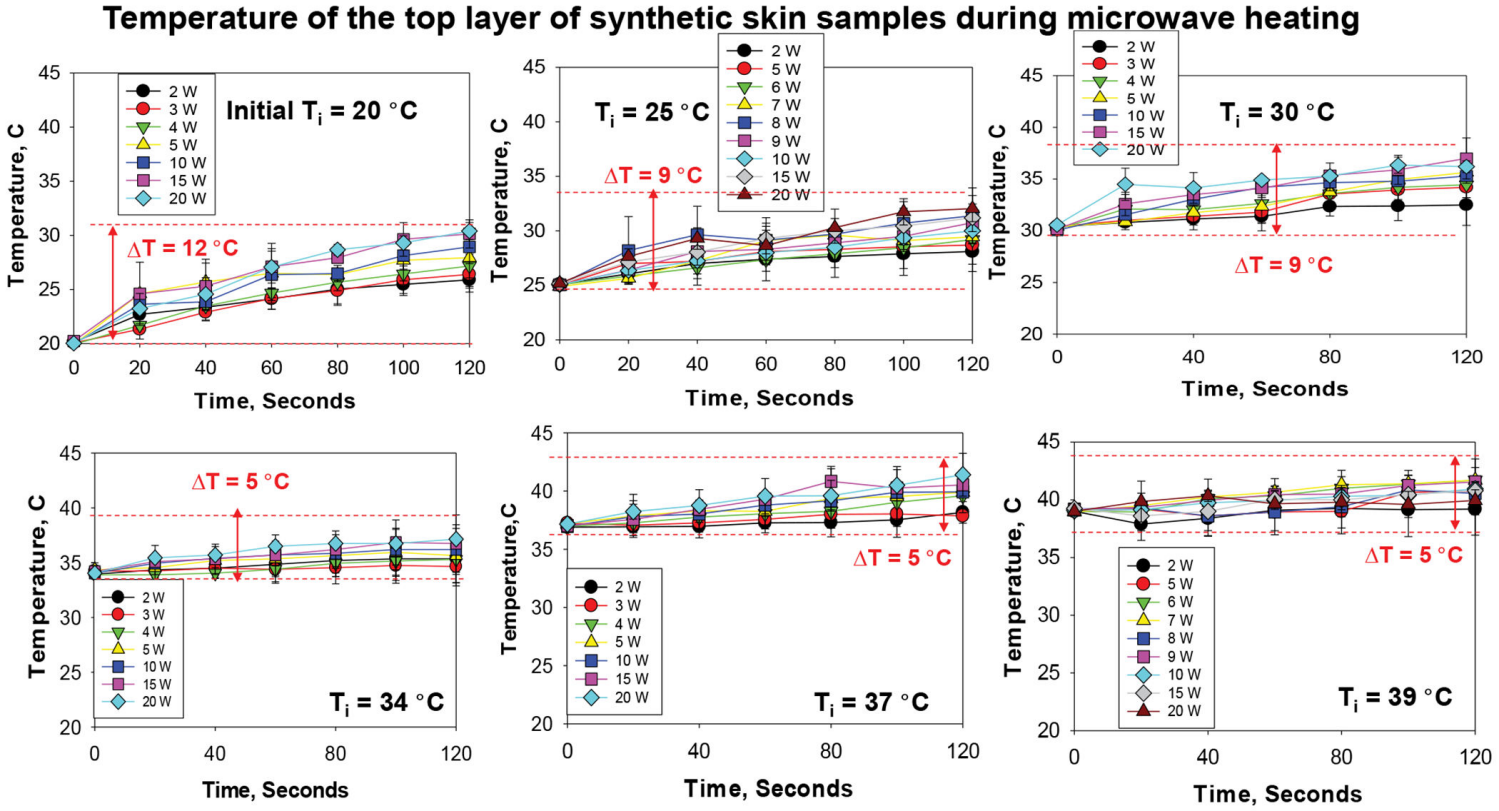
Change in temperature of synthetic skin during microwave heating to assess potential changes in surface area (2 W-20 W) up to 120 sec. Initial temperature of the synthetic skin was adjusted to 20 °C, 25 °C, 30 °C, 34 °C, 37 °C and 39 °C. Room temperature was ~25 °C at the time of experiments.

In addition to visual inspections of damage to the synthetic skin samples, temperature measurement of the top layers of the synthetic skin samples provided greater insight into damage to the samples during microwave heating. Since we seek to develop the MAMAD technique as an alternative treatment for gout and other crystal deposition diseases that would not disrupt the specific biologically functions of the human body, it is important to monitor the changes in temperature during microwave heating of the synthetic skin samples. According to Figure 10 [31], at 20 °C, the largest temperature change of ~12 °C after 120 seconds of microwave heating is observed. As the initial temperature is raised to 39 °C the temperature change ranges between ~5 to 9 °C. These observations can be attributed to the system (synthetic skin samples, Au NPs and L-alanine) establishing thermal equilibrium through heat transfer with the surroundings (air at 25 °C). Microwave heating of the system at 20 °C results in heat transfer to the system from the surroundings until the temperature of the system and the surroundings is at the same temperature. As microwave heating is continued and the temperature of the system is higher than that of the surroundings, heat transfer occurs from the system to the surroundings. As the initial temperature of the synthetic skin samples is increased above 25 °C, heat transfer occurs from the system to the surroundings. Moreover, theoretical calculations based on finite-difference time-domain method predict a 10 °C increase in temperature for synthetic skin heated at 10 W for 120 seconds. The experimental results validate these predictions.

**Figure 11:**
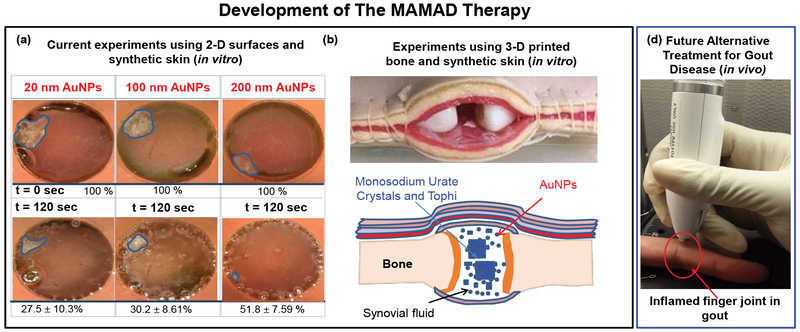
Potential Future Application of the MAMAD Therapy (a) The proof-of-principle demonstration of the MAMAD technique for the potential treatment of advanced gout using 10 W power level and L-alanine as a model tophi, synthetic skin on 2-D surfaces; (b) Comparison of MAMAD technique with various sizes of Au NPs for 120 s; (c) 3-D printed bones (*in vitro*). The size of the L-alanine crystals was reduced using the MAMAD technique with Gold Nanoparticles (Au NPs). Synthetic skin samples were kept at 37 °C; (d) Potential future application of the MAMAD technique to the treatment of gout at various stages of the diseases by medical professionals.

Figure 11A shows the results of the MAMAD technique when applied to the decrystallization of a model crystal (L-alanine) placed under synthetic skin samples in the presence of various sizes of Au NPs. It is important to note that the 10 W power level is used due to the observation that negligible damage to the synthetic skin samples in the presence of Au NPs occurs at this wattage. That is, the use of Au NPs affords for additional durability to the synthetic skin when exposed to microwave heating beyond 7 W. Figure 11A shows Au NPs of varying sizes can be used with the MAMAD technique. A minimum 48% reduction in the surface area of L-alanine crystals was observed for all Au NPs sizes. In addition, we have further developed our joint model by introducing 3D printed bones as shown in Figure 11B, where we aim to obtain a more holistic view of the MAMAD technique and its potential applications in the treatment of gout and other crystal deposition diseases. We hypothesize that the MAMAD technique can possibly be implemented in current treatment of gout patients in the future Figure 11C.

## Conclusions and Outlook

In this review article, we presented a summary of the past and current advancements achieved in the application of the MAMAD technique for the rapid breakdown of organic crystals (UA, L-alanine and MSUM) and the effects of microwave heating on synthetic skin samples and in artificial joint models. The studies to date yielded several conclusions for the use of the MAMAD technique as an alternative approach to potential treatment of crystal deposition diseases in terms of:

### Efficiency

The MAMAD technique can be applied to crystals of varying sizes (UA ~10-100 μm, MSUM and L-alanine ~300-4,500 μm) on planar surfaces. As we further developed more complex experimental models (skin and pouch were used to encase the crystals) to mimic gout conditions, the use of the MAMAD technique yielded significant decrease in crystal surface area, count, and size in these models. Au NPs in the size range of 20-200 nm can be employed with the MAMAD technique.

### Rapidity

We demonstrated the proof-of-principle use of the MAMAD technique for potential use in mammals by implementing a solid state medical microwave source. Significant decrystallization of crystals can be obtained in as little as 120 seconds.

### Repeatability

The MAMAD technique is highly repeatable due to the stability of the microwave source afforded by solid state microwave technology and stability of Au NPs in biological media during microwave heating.

### Safety

Based on our results obtained *In vitro*, where microwave heating of biological samples using a solid-state microwave source is carried out, it is reasonable to assume that the MAMAD technique will cause minimal damage to the biological functions in humans when employed within the parameters described here. In this regard, further studies using rat models are needed.

Our laboratory is developing a more advanced model that can provide a better representation for the synovial joints in the human body. Subsequently, we are implementing the use of 3D printing technology to create bones, which can then be combined with synthetic skin samples and pouch to create an artificial joint. Additionally, we have created hard tophi masses *in vitro*, which is similar to that formed in the human body. We plan to investigate whether the MAMAD technique can be used to reduce the size of tophi masses *in vitro* and in rat models so that the effects of the technique *in vivo* can be examined. The results of these experiments will be reported in due course.

## Abbreviations

MAMAD: Metal-Assisted and Microwave-Accelerated Decrystallization
UA: Uric Acid
MSUM: Monosodium Urate Monohydrate
SEM: Scanning Electron Microscopy
Au NPs: Gold Nanoparticles
Ag NPs: Silver Nanoparticles
